# Marginal Eyeliner Use and Meibomian Gland Function

**DOI:** 10.3390/jcm15072616

**Published:** 2026-03-29

**Authors:** Mariam Alkawally, Rachelle J. Lin, Corina van de Pol, Alan Sasai, Andrew Loc Nguyen, Jerry R. Paugh

**Affiliations:** 1 Independent Researcher, Calgary, AB T2V 4J2, Canada; dr.alkawally@gmail.com; 2 Southern California College of Optometry, Marshall B. Ketchum University, Fullerton, CA 92831, USA; rlin@ketchum.edu; 3 School of Optometry, Highpoint University, Highpoint, NC 27260, USA; cvandepol@highpoint.edu; 4 Independent Researcher, Garden Grove, CA 90620, USA; alan.s.sasai@gmail.com; 5 Department of Mathematics, California State University, Fullerton, CA 92831, USA

**Keywords:** makeup, eyeliner, meibomian gland excreta, meiboscore

## Abstract

**Background/Objectives:** To investigate whether chronic cosmetics use near or directly on the eyelid margin contributes to tear film instability and meibomian gland dysfunction. **Methods:** Subjects were enrolled in one of three groups: those who rarely wear makeup (No-M), those who wear it frequently but only outside the eyelid margin (Min-M), and those who wear it frequently and directly on the eyelid margin (W-M). Subjects were assessed for dry eye signs and symptoms by a masked examiner. Lipid layer thickness (LLT), tear meniscus height, meibomian gland excreta grade, number of glands secreting, corneal and conjunctival staining and tear breakup time were assessed. **Results:** 10 No-M, 18 Min-M, and 21 W-M subjects completed the study. Average fluorescein breakup time was 4.6 s in each group (*p* = 0.839, 1-way ANOVA). There were higher scores (worse findings) in the marginal eyeliner sample for symptoms (modified Schein, OSDI, SPEED), Oxford and total NEI staining and lower lid meibomian secretions. The W-M group demonstrated a statistically significant increase in the meibomian gland excreta grade (a worsening) compared to the No-M group (mean grades 1.2 and 0.55 respectively; Tukey test, adjusted *p* < 0.05, 95% CI 0.055–1.187). LLT, tear breakup time, eyelid marginal signs, and meibomian gland dropout had no differences among groups. **Conclusions:** Eyeliner wear both outside and on the eyelid margin demonstrated increased ocular staining and decreased gland excretion quality, compared to non-makeup users. The meibomian gland excreta decrement may lead to worsening meibomian gland function and potentially glandular atrophy over time.

## 1. Introduction

Cosmetics have been used around the ocular adnexa (eyelids, lashes and eyelid margins) for millennia and their use continues today. Despite several reviews enumerating the actual and potential effects of ocular cosmetic use [1,2,3], there are relatively few reports of the direct effects on the tear film and ocular surface from either short-term cosmetics use or the chronic effects of consistent makeup application.

Ng et al. [4] in a pilot study demonstrated migration into the tear film from acute application of eyeliner both inside and outside the lash line, with greater migration from the application inside the lash line. The number of cosmetic particles became negligible in both scenarios after about two hours. Ng [5] also examined eyeliner effect inside and outside the lash line in a crossover trial. After one day, there were no clinically nor statistically significant differences in the ocular surface parameters of either lash line location eyeliner application [5]. After 7 days of daily eyeliner use, the lipid layer improved (thickened) slightly in the sample using eyeliner inside the lash line although the OSDI score increased, but not to a dry eye level [5]. It seems likely that eyeliner use within the lash line may have the most significant effect on meibomian gland function by potentially blocking orifices and increasing direct cosmetic migration to the pre-corneal tear film.

Prabhasawat and co-workers [6] examined chronic eyeliner use in younger women. They selected their sample based on normal OSDI scores (<13) and separated them into two groups: an eyeliner use group (either anterior to or posterior to the lash line) and a non-eyeliner use control group. They found significant decreases in tear stability, meibomian secretion quality and expressibility, and meibomian gland loss in the eyeliner group compared to the control. Limitations of this study [6] included apparent eyeliner application at the time of examination, which may have affected tear stability, the lack of results separated by anterior vs. posterior lash line eyeliner use, and forceful expression of the meibomian glands, which would be a non-physiological expression. Moreover, selecting only subjects without dry eye symptoms may have biased the sample as chronic eyeliner use may induce dry eye.

The purpose of this study was to examine tear film and meibomian gland parameters in a group of young, chronic cosmetics users differentiated by eyeliner use inside vs. outside the lash line, compared to those who used no makeup at all.

## 2. Materials and Methods

### 2.1. Study Design

This was a prospective, cross-sectional, investigator-masked observational pilot study with a principal outcome measure of lipid layer thickness (LLT) measured using the LipiView^®^ II imaging instrument (Johnson and Johnson Vision Care, Jacksonville, FL, USA). Secondary outcome measures included symptoms, tear meniscus height (TMH), eyelid marginal signs, fluorescein tear breakup time (TBUT), ocular surface staining, meibum secretion score, and meibography. The study was conducted from October 2018 to January 2020.

### 2.2. Subjects

This study comprised a convenience sample, recruited from staff, students, and faculty of local universities. Major inclusion criteria were female subjects aged 18–35, willing to discontinue topical artificial tears and any facial makeup use on the day of the examination and best corrected visual acuity of 20/25 or better. Soft lens wearers were included so long as lenses were not worn on the day of examination, but no rigid or scleral lens wearers were included. Major exclusion criteria included history of any isotretinoin use, prior diagnosis of Sjogren’s syndrome or other autoimmune conditions, a Schirmer I test result < 5.0 mm wetting during examination, prior diagnosis of seborrhea sicca, seborrheic dermatitis or acne rosacea, history or evidence of active ocular allergy or infection, evidence of greater than mild eyelid deformity (e.g., ectropion, entropion, ptosis), refractive or intraocular surgery within the previous 12 months, individuals with diabetes or use of systemic tetracyclines within the previous 30 days, and individuals with punctal plugs. Pregnant or lactating females were excluded as were those using systemic tear-influencing medications (e.g., antihistamines) unless they had been on a stable dosing regimen for at least 30 days.

The study adhered to the tenets of the Declaration of Helsinki and all subjects provided written, informed consent following explanation of the nature and possible consequences of the study prior to the administration of study procedures. The Institutional Review Board of Marshall B. Ketchum University approved the study protocol and study conduct.

### 2.3. Recruitment and Classification of Subjects

Unmasked research assistants administered a makeup history questionnaire that was adapted from that used in Dr. Alison Ng’s doctoral project [7]. The survey determined the frequency, location and type of makeup used. Enrolled subjects were grouped into three categories: (1) those who never or very rarely (i.e., ≤twice per month) wear makeup (No-M), (2) those who wear it frequently (≥4 times per week) but only outside the eyelid margin (Min-M), and (3) those who frequently (≥4 times per week) wear it directly on the eyelid margin posterior to the lash line (W-M). For inclusion, subjects’ makeup-wearing habits had to be consistent for a minimum of 1 year. The examining clinician was masked relative to the makeup classification until data analysis was complete.

### 2.4. Clinical Study Procedures

Subjects presented for clinical examination with no makeup of any kind nor artificial tear use in the preceding 12 h period. Testing progressed from least to most invasive and included both eyes. Medical and contact lens history were queried for inclusion suitability, then symptom questionnaires (modified Schein, OSDI and SPEED), lipid layer thickness (Lipiview II; Johnson & Johnson Vision, Jacksonville, FL, USA), tear meniscus height (biomicroscope graticule), general biomicroscopy and eyelid marginal sign grading (present or not, 0–6 score; orifice metaplasia, vascularity/brush marks, capped glands, ridging, margin irregularity and margin displacement) were undertaken.

Tear stability was assessed as tear breakup time (TBUT) using a micropipette with unpreserved liquid sodium fluorescein (2.0 μL of 1.0% wt./vol concentration) instilled into each eye, with three natural blinks, and use of a yellow barrier filter [8]. A mean of three stability measurements was obtained for each determination. Corneal staining followed TBUT determination within 4–8 min [9] and was graded using both the Oxford and NEI systems. Conjunctival staining was undertaken using lissamine green (strips, wetted and excess shaken off) also using the Oxford and NEI systems.

Meibomian gland function was assessed across the entire lower eyelid using a Meibomian Gland Evaluator (MGE, Johnson and Johnson Vision Care, Jacksonville, FL, USA) [10]. An average secretion score for the expressible glands was determined using the Bron scale [11,12] modified with 0.1 scale unit increments. Meibomian Glands Yielding Liquid Secretion (MGYLS) [10] was determined across the entire lower eyelid. The Schirmer I test was conducted without anesthetic and the strip was moved at 2 min if no wetting was observed [13]. The amount of strip wetting in five minutes was recorded.

Meibography was undertaken using the LipiView II (Johnson and Johnson Vision Care, Jacksonville, FL, USA) which imaged upper and lower eyelids. The total meiboscore was determined by summing the individual eyelid scores (each on a 0–3 scale [14,15]) for a total eye score scale of 0–6.

### 2.5. Sample Size Estimate

An a priori sample size estimate was undertaken using LLT. For three sample (No-M, Min-M, W-M) groups, we assumed a 1-way ANOVA comparison of groups, a Type I error level of 0.05, and a Type II error level of 0.20 (80% power). Using LLT data from our laboratory (mean value of 60 nm LLT with a standard deviation of 17 nm in 25 young subjects), and an arbitrary clinical meaningful difference of 17 nm (~28% of mean), the sample size for each arm was 21 subjects.

### 2.6. Statistical Analysis

Statistical analysis was undertaken using Minitab, Version 18 (Minitab LLC, State College, PA, USA). The principal outcome parameter was lipid layer thickness (LLT) in nm in the three groups. For each parameter, the general linear model (GLM) was used to identify whether possible confounders (contact lens wear status and race) may have influenced the result. Factors with large, non-significant *p*-values from the initial ANOVA were removed and the GLM repeated. If factors in the final GLM were significant from the second ANOVA, Tukey simultaneous tests for differences in means were conducted for pairwise comparisons.

## 3. Results

Of the 219 subjects screened, 54 satisfied the initial qualification criteria, and data from 49 subjects were used for this study: 10 No-M (minimal makeup frequency), 18 Min-M (makeup outside lash line, ≥4× per week), and 21 W-M (eyeliner behind lash line ≥ 4× per week).

Subjects ranged in age to 35 from 21 years, with an average age of 25.1 years. The mean age for the No-M group was 23.9 ± 1.4 years, 25.0 ± 3.6 years for the Min-M group, and 25.8 ± 3.0 years for the W-M group. There was no significant difference in age among the three groups (1-way ANOVA, *p* = 0.280).

The general linear model was applied to assess whether differences in contact lens wear status or race between the groups may have influenced any of the outcome measures; neither factor had a significant influence on the results (*p* > 0.2 and *p* > 0.7, respectively for all parameters). The types of makeup used in the two makeup groups (Min-M and W-M) are summarized in Table 1. Multiple subjects in each group reported using multiple eyeliner types (3 out of 18 in the Min-M group, 9 out of 21 in the W-M group). For subjects reporting a single type of eyeliner used, the sample was too small for statistically meaningful analysis.

Dry eye symptomatology with the modified Schein, OSDI and SPEED surveys is summarized in Table 2. Although there was no statistical difference among the three groups, the No-M sample had lower scores on all questionnaires and the trend was toward greater scores (worse symptoms) in the Min-M and W-M samples.

The global tear film and ocular surface data are summarized in Table 3. Lipid Layer Thickness (LLT, in nm), the principal outcome measure, for the No-M, Min-M and W-M groups were 64.5 ± 17.4, 59.6 ± 21.4, and 63.1 ± 21.4 respectively) and these were not different (*p* = 0.673, 1-way ANOVA). On average, subjects from all three groups had at, or slightly greater than 60 nm LLT, a level suggestive of dry eye values [16].

All three makeup groups demonstrated fluorescein breakup times that averaged 4.6 s, below the cut-point for dry eye diagnosis [17]. However, the No-M group had no other indicators of dry eye (Table 3 and Table 4), and were considered to have normal ocular surface status. There were no statistically significant differences among the 3 groups for TBUT measurements (*p =* 0.673, 1-way ANOVA).

Both staining scores, NEI and Oxford, were higher in the two makeup-wearing groups, and significantly lower in the No-M group compared to the Min-M group (*p =* 0.021 and 0.018, respectively, Tukey pairwise comparison; Table 3). The NEI and Oxford average scores for the two makeup groups were close to the diagnostic cut-point for evaporative dry eye [18].

The meibomian gland-associated data are summarized in Table 4. There were no statistically significant differences between the three groups (No-M, Min-M and W-M) for lower eyelid marginal signs, lower eyelid MGYLS, or combined upper and lower eyelid meiboscore. However, the W-M group had a significantly higher average lower eyelid expression grade (0–3 Bron scale; [11] of 1.1 ± 0.8) compared to the No-M group (0.5 ± 0.4 grade; *p* = 0.029, Tukey pairwise comparison), whereas Min-M vs. W-M and No-M vs. Min-M were not statistically significantly different (*p* > 0.05 for both; Table 4).

## 4. Discussion

This study was a preliminary investigation into the possible effects of chronic (≥1 year) eyeliner use on the ocular surface and meibomian gland structure and function, differentiated by whether and where the makeup was applied. The eyeliner application location (i.e., outside or inside the lid margin, split by the lash line) was important as was masking of the makeup type and the gentle (i.e., physiological) expression to assess meibomian gland secretions.

The results demonstrate that several tear film and ocular surface parameters trended toward dry eye (e.g., symptoms and staining) although other measures did not (e.g., LLT, marginal signs, MGYLS and meibomian gland morphology). The most significant finding was that the meibomian gland secretion quality, with an average grade of 1.1 ± 0.8 (Table 4), was elevated to dry eye levels [12,18] in the makeup behind the lash line group; i.e., greater than grade 1.0 (on the 0–3 Bron scale [12])**,** compared to those who wear makeup outside the lash line or those who do not wear any makeup.

The possible explanations for the increased (worse) secretion grade in the marginal eyeliner group are that (1) it could have been coincidental in this limited sample, or that (2) the frequent use of eyeliner, particularly the pencil (waxy) type, may have blocked the orifices frequently enough to obstruct the excretion of meibum. If meibum excretion is obstructed to a sufficient degree, it may become stagnant, eventually leading to acinar atrophy [19]. These secretion changes appear real in eyeliner users since secretion score elevation was also observed in the forceful gland expression by Prabhasawat et al. [6].

A curious finding was the statistical significance in the two staining parameters (NEI and Oxford) for the No-M group compared to the Min-M group (Table 4). The greatest means and medians were found for the Min-M group, and illustrated significance vs. No-M. However, the W-M group, expected to have the greatest staining of all three groups, did not achieve significance vs. the No-M group; we believe that this is not a true effect, but rather a function of the limited sample size and a reason to repeat this study with larger numbers of subjects. A possible rationale as to why the ocular surface parameters were not worse in the makeup groups than found herein might be due to the mechanical activity of applying and removing makeup and/or the debridement performed in the process of marginal eyeliner application. These actions are akin to the massage recommended in managing meibomian gland dysfunction that have been widely demonstrated to improve ocular surface parameters.

### Limitations of the Study and Future Research

Limitations of this study include the modest sample size and not controlling for the exact type and brand of eyeliner used. Future research requires greater enrollment of eyeliner users and stratification by the type of eyeliner, particularly the use of the waxy pencil type. Moreover, our subjects were limited to the age range of 18–35 years, which was deliberate in order to eliminate confounding causes of dry eye such as age and hormonal changes. Studies in older and younger age groups, with attention to length of cosmetics use and hormonal status, will be important to elucidate the full effect of makeup use on the ocular surface. A broader age demographic may determine whether eyeliner use exacerbates the normal aging changes in the ocular surface and meibomian gland function.

## 5. Conclusions

Eyeliner wear, both outside and on the eyelid margin, has a negative effect on ocular surface parameters such as symptoms, staining, and meibum excreta quality compared to no eyeliner wear. Additional research with larger sample sizes and a wider age range of women is necessary to confirm these apparent trends and to determine the long-term impact of different types of eyeliner wear on meibomian gland function.

## Figures and Tables

**Table 1:** Eyeliner type, makeup-using groups.

	Min-M (n = 17) *	W-M (n = 20) *
**Multiple Types**	3	9
**Powder**	1	0
**Pencil**	3	4
**Liquid**	10	5
**Gel**	0	2

* One subject from each of the Min-M and W-M groups did not report the type of eyeliner used.

**Table 2:** Summary of symptom questionnaire scores.

Questionnaire (Scale Range)	Subject Group	Mean ± SD (Median)	1-Way ANOVA *p*-Value	Tukey Pairwise Comparison		
				No-M vs. Min-M	Min-M vs. W-M	No-M vs. W-M
**Modified Schein** (0–24)	**No-M** (n = 10)	3.8 ± 2.7 (3.5)	*p* = 0.290	N/A	N/A	N/A
	**Min-M** (n = 18)	5.7 ± 3.4 (5.5)				
	**W-M** (n = 21)	5.3 ± 2.9 (5.0)				
**OSDI** (0–100)	**No-M** (n = 10)	8.1 ± 8.2 (4.17)	*p* = 0.326	N/A	N/A	N/A
	**Min-M** (n = 18)	13.5 ± 10.8 (12.5)				
	**W-M** (n = 21)	11.0 ± 8.12 (8.3)				
**SPEED** (0–28)	**No-M** (n = 10)	4.5 ± 4.6 (3.5)	*p* = 0.377	N/A	N/A	N/A
	**Min-M** (n = 18)	6.8 ± 4.2 (6)				
	**W-M** (n = 21)	6.3 ± 5.3 (7)				

**Table 3:** Summary of Global Clinical Data.

Parameter (Range)		Subject Group	Mean ± SD (Median)	1-Way ANOVA *p*-Value	Tukey Pairwise Comparison *p* (95% CI)		
					No-M vs. Min-M	Min-M vs. W-M	No-M vs. W-M
**Lipid Layer Thickness (LLT)** (0–100+ nanometers)		**No-M** (n = 10)	64.5 ± 17.4 (60.0)	*p* = 0.673	N/A	N/A	N/A
		**Min-M** (n = 18)	59.6 ± 21.4 (55.0)				
		**W-M** (n = 21)	63.1 ± 21.4 (64.0)				
**Tear Breakup Time (TBUT, s)**		**No-M** (n = 10)	4.6 ± 1.4 (4.7)	*p* = 0.839	N/A	N/A	N/A
		**Min-M** (n = 18)	4.6 ± 2.2 (4.4)				
		**W-M** (n = 21)	4.6 ± 2.2 (4.0)				
**Tear Meniscus Height (TMH, mm)**		**No-M** (n = 10)	0.3 ± 0.09 (0.3)	***p*** ** = 0.046**	***p* = 0.037** (0.004–0.161)	*p* = 0.366 (−0.028–0.100)	*p* = 0.318 (−0.123–0.030)
		**Min-M** (n = 18)	0.2 ± 0.09 (0.2)				
		**W-M** (n = 21)	0.3 ± 0.07 (0.3)				
**Staining**	**Total NEI** (0–33)	**No-M** (n = 10)	3.8 ± 4.2 (2.5)	***p*** ** = 0.027**	***p* = 0.021** (−7.19 to −0.51)	*p* = 0.343 (−4.34–1.15)	*p* = 0.230 (−1.03–5.53)
		**Min-M** (n = 18)	7.3 ± 4.1 (7.5)				
		**W-M** (n = 21)	6.0 ±3.1 (5.0)				
	**Total Oxford** (0–15)	**No-M** (n = 10)	5.6 ± 2.5 (5.0)	***p*** ** = 0.023**	***p* = 0.018** (−4.79 to −0.39)	*p* = 0.528 (−2.61–1.00)	*p* = 0.124 (−0.38–3.93)
		**Min-M** (n = 18)	8.1 ± 2.5 (7.5)				
		**W-M** (n = 21)	7.3 ± 1.8 (7.0)				

**Table 4:** Summary of meibomian gland-specific data.

Parameter (Range)	Subject Group	Mean ± SD (Median)	1-Way ANOVA *p*-Value	Tukey Pairwise Comparison *p* (95% CI)		
				No-M vs. Min-M	Min-M vs. W-M	No-M vs. W-M
**Total Lid Marginal Signs** (0–6)	**No-M** (n = 10)	3.0 ± 0.5 (3.0)	*p* = 0.424	N/A	N/A	N/A
	**Min-M** (n = 18)	3.3 ± 0.9 (3.0)				
	**W-M** (n = 21)	3.0 ± 0.8 (3.0)				
**Total Lower Lid MGYLS** (0–24)	**No-M** (n = 10)	14.9 ± 6.8 (16.5)	*p* = 0.796	N/A	N/A	N/A
	**Min-M** (n = 18)	16.4 ± 7.2 (18.5)				
	**W-M** (n = 21)	16.0 ± 6.5 (18.0)				
**Average Lower Lid Expression Grade** (0–3, in 0.1 scale unit increments)	**No-M** (n = 10)	0.5 ± 0.4 (0.5)	***p*** ** = 0.016**	*p* = 0.788 (−0.739–0.042)	*p* = 0.058 (−0.013–0.940)	***p* = 0.029** (0.055–1.187)
	**Min-M** (n = 18)	0.6 ± 0.6 (0.5)				
	**W-M** (n = 21)	1.1 ± 0.8 (0.8)				
**Total Meiboscore** (0–6)	**No-M** (n = 10)	0.8 ± 0.8 (0.5)	*p* = 0.955	N/A	N/A	N/A
	**Min-M** (n = 18)	0.7 ± 0.8 (0.5)				
	**W-M** (n = 21)	0.8 ± 1.3 (0.5)				

## Data Availability

The original contributions presented in this study are included in the article. Further inquiries can be directed to the corresponding author.

## Abbreviations

The following abbreviations are used in this manuscript:

No-M	Rarely wear makeup
Min-M	Wear makeup frequently, but only outside the eyelid margin
W-M	Wear makeup frequently and directly on the eyelid margin
